# Tissue-Specific Differential Distribution of Cell Wall Epitopes in *Sphagnum compactum* and *Marchantia polymorpha*

**DOI:** 10.3390/ijms26083602

**Published:** 2025-04-11

**Authors:** Ioannis-Dimosthenis S. Adamakis, Penelope Sotiriou, Natalia Ntanou, Jessica M. Nelson, Eleni Giannoutsou

**Affiliations:** 1 Section of Botany, Department of Biology, National and Kapodistrian University of Athens, 15784 Athens, Greece; penysotiriou@biol.uoa.gr (P.S.); sbi1900068@uoa.gr (N.N.); egianno@biol.uoa.gr (E.G.); 2 Natural History Museum, University of Oslo, 0562 Oslo, Norway; jessica.nelson@nhm.uio.no

**Keywords:** bryophytes, cell wall composition, *Sphagnum compactum*, *Marchantia polymorpha*, polysaccharides

## Abstract

Bryophytes, or non-vascular plants, provide valuable models for studying plant adaptation to land, as their physiology differs significantly from that of vascular plants. This study examines the cell wall structure of bryophytes, focusing on the tissue-specific distribution of cell wall epitopes in *Sphagnum compactum* (a peat moss) and *Marchantia polymorpha* (the model liverwort) using specific stains and immunolabeling techniques. In *S. compactum*, chlorocysts and hyalocysts exhibit distinct polysaccharide compositions, with methylesterified and demethylesterified homogalacturonans, arabinans, and hemicelluloses contributing to water retention, structural integrity, and photosynthetic efficiency. In contrast, *M. polymorpha* demonstrates a simpler yet polarized distribution of homogalacturonans, arabinans, mannans, and xyloglucans, with arabinogalactan proteins uniquely localized in rhizoids, improving their flexibility and anchorage to the substrate. Cellulose was uniformly distributed throughout all tissues in both bryophytes, while crystalline cellulose was only faintly observed. These findings highlight how cell wall adaptations contribute to ecological specialization, providing insights into the evolutionary innovations that enable bryophytes to thrive in terrestrial environments.

## 1. Introduction

The transition of plant ancestors from aquatic to terrestrial environments approximately 450 million years ago necessitated profound changes in cell wall structure and composition [1]. These adaptations were crucial for overcoming challenges such as desiccation, UV radiation, and the need for structural support. Key innovations included enhanced water regulation, facilitated by hydrophilic hemicelluloses like mannans [2], and the development of a protective cuticle barrier [3]. Additionally, plants evolved UV protection mechanisms through phenolic compounds embedded in cell walls, vacuolar phenolics, and the cuticle itself [4]. The synthesis of lignified secondary walls further enabled vertical growth and efficient water transport, marking a significant evolutionary milestone for tracheophytes [5]. These advancements in cell wall biology laid the foundation for the diversification of land plants [6].

Bryophytes, including mosses and liverworts, exhibit distinct adaptations to terrestrial life compared to their vascular counterparts, maintaining a greater dependence on water availability for reproduction and growth [7]. Despite their small size, bryophytes play essential ecological roles, acting as pioneers in soil formation, regulators of water cycles, and contributors to carbon sequestration [8,9]. They serve as ecological engineers by creating microhabitats that support, for example, diverse microbial communities [10]. Furthermore, their cellular biology provides valuable insights into plant function and serves as a simpler model for studying the evolution of complex traits in angiosperms [11]. In bryophytes, the types of tissue present are few and the cellular pathways much simpler. For example, the model liverwort, *Marchantia polymorpha*, has a minimal set of homologs for the genes involved in the auxin signaling pathway: six as compared to the 63 in the angiosperm model, *Arabidopsis thaliana* [12]. Investigating the physiological and ecological adaptations of bryophytes enhances our understanding of plant evolution and informs conservation efforts, particularly in the context of climate change and habitat degradation [13,14].

Research on the cell wall composition of the green lineage has provided insights into bryophyte adaptations [15]. Cell wall composition has been shown to influence both cell wall thickness and photosynthetic rates in certain mosses [16], underscoring the physiological importance of studying cell wall properties across bryophytes. Mannans, which serve as the primary hemicelluloses in charophytes, were detected in bryophytes through paper chromatography following acid hydrolysis of cell walls [17]. However, bryophyte mannans exhibit higher mannose levels than those of vascular plants, suggesting a divergence that may be associated with differing functional requirements. Similarly, moss and liverwort xyloglucans differ structurally from those in flowering plants, containing galacturonic acid as revealed by mass spectrometry [18], which may reflect adaptations to their specific environmental conditions. Xyloglucan, a vital structural component, was identified in hornworts, mosses, and liverworts, with driselase digestion confirming its presence through isoprimeverose detection. Uronic acid composition also varied, with bryophytes showing higher glucuronic acid levels than vascular plants. Notably, *Anthoceros* contained a unique glucuronic acid-α(1→3)-galactose repeat unit, absent in other species, highlighting biochemical differentiation within bryophytes [17]. Additionally, the cell walls of mosses and liverworts are enriched with phenolic compounds such as p-coumaric and ferulic acids, which contribute to UV protection and structural stability [4], enhancing their resilience in exposed habitats. The absence of mixed-linkage glucan (MLG) in algae and bryophytes, despite the presence of MLG-related polysaccharides in *Ulva lactuca* and *Lophocolea bidentata*, suggests independent adaptation strategies [17]. These findings highlight the evolutionary diversification of cell wall composition across the green lineage, with bryophytes exhibiting distinct polysaccharide profiles compared to both charophyte algae and vascular plants [17].

The *Sphagnum* genus, apart from exhibiting significant intra- and interspecific variation in nucleotide sequences, varies in physiology, morphology, net production, and carbon accumulation (peat formation). With many species, diverse mating systems, and distinct patterns of niche differentiation, the *Sphagnum* lineage serves as an exceptionally valuable addition to *Physcomitrium* (*Physcomitrella*) *patens* [19] and *Ceratodon purpureus* as a moss model for genomic research [20,21]. It is also of particular interest for cell wall studies because of its remarkable resistance to decomposition. Polysaccharide research in *Sphagnum* mosses, which began in the 1950s and accelerated with the identification of D-lyxo-5-hexopyranuronic acid in 1983, has revealed that their cell walls are largely composed of cellulosic and pectin-like structures, with key sugars such as D-galacturonic acid and L-rhamnose [22]. Of particular interest are the moss’s hyaline cells, which enable it to absorb up to 25 times its dry weight in water [23]. The specific cell wall components of Sphagnum mosses collectively enable substantial water retention, particularly within specialized hyaline cells [24]. Polyphenolic polymers, such as sphagnic acid, protect cell wall polysaccharides from degradation [25], while sphagnan, a pectin-like carbohydrate, further safeguards organic material from microbial decomposition [26]. Additionally, *Sphagnum* cell walls exhibit a high cation exchange capacity, contributing to the acidification of peatlands and their unique ecological dynamics [27].

*Marchantia polymorpha* serves as a key model organism for plant biology, offering valuable insights into the development and morphology of non-vascular land plants. It has been a cornerstone of biological research since the 18th century, with its use experiencing a significant revival in recent decades [28]. This resurgence is driven by its suitability for genomic and genetic research, particularly in studying the physiological, developmental, and evolutionary aspects of land plants. Its advantages as a model system include simple cultivation, worldwide availability, ease of crossing, straightforward genetics, and access to advanced tools such as efficient transformation techniques, genome editing, and comprehensive genomic resources [28,29,30]. The cell walls of *M. polymorpha* display remarkable flexibility, a critical adaptation for its flat thallus structure. This flexibility is attributed to a high pectin content, providing porosity, elasticity, and adhesion [31]. The cell wall proteome of *M. polymorpha* reveals distinct evolutionary traits, including specialized arabinogalactan proteins absent in angiosperms [32]. Moreover, the thallus contains air chambers lined with specialized cell walls that facilitate efficient gas exchange, promoting photosynthesis [33]. Beyond structural roles, *Marchantia* cell walls contain secondary metabolites such as flavonoids, which enhance defense mechanisms against herbivores and pathogens. For instance, auronidin flavonoid pigments form polymers that strengthen cell walls and bolster protection [34].

Although techniques such as hydrolytic analyses and residual material weighting have yielded valuable information about the components of bryophyte cell walls [15,17,35], there is still a need for more detailed structural insights into the specific characteristics of cell walls across different tissues in mosses and liverworts. Previous studies have typically focused on specific cell types, such as hyaline cells, elaters [36], pseudo-elaters, spores [37,38], placental cells [39], leptoids [40], or hydroids [41], leaving gaps in our understanding of the broader cell wall characteristics across diverse tissue types. To fill this gap, this study offers a comprehensive analysis of the distribution of various cell wall epitopes in two bryophytes: the peat moss *Sphagnum compactum* and the model liverwort *Marchantia polymorpha*. This study investigates and compares the cell wall structures and compositions of *S. compactum* and *M. polymorpha*, focusing on the distribution of key polysaccharides and proteins. Immunocytochemical/staining techniques were used to examine all gametophyte tissues of each species for glucans (crystalline cellulose and callose) homogalacturonans (HG), arabinans, xyloglucans, mannans, and arabinogalactan proteins (AGPs). By uncovering the unique cell wall characteristics of *Sphagnum* and *Marchantia*, this research could shed light on how these adaptations support their functional roles in various terrestrial habitats.

## 2. Results

### 2.1. Spagnum compactum

**Figure 1:**
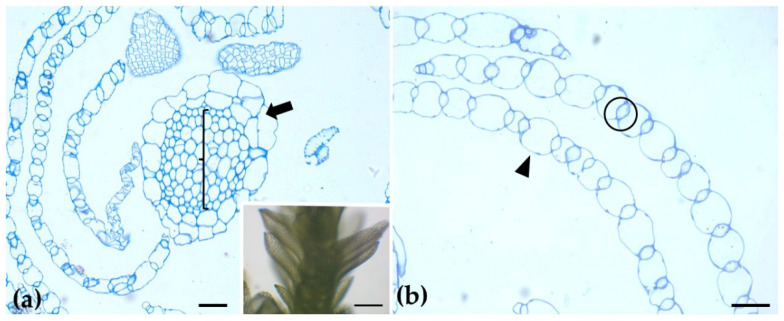
Transverse sections of the gametophyte stem (**a**) and branch leaves (**b**) stained with toluidine blue. The stem’s epidermis consists of hyaline cells (arrow), while the central cylinder is marked by a bracket. Branch leaves comprise alternating hyaline cells (arrowhead) and photosynthetic cells (circle). Inset: stem and branch leaves of *S. compactum*. Scale bars: (**a**,**b**): 20 μm; Inset: 0.5 mm.

**Figure 2:**
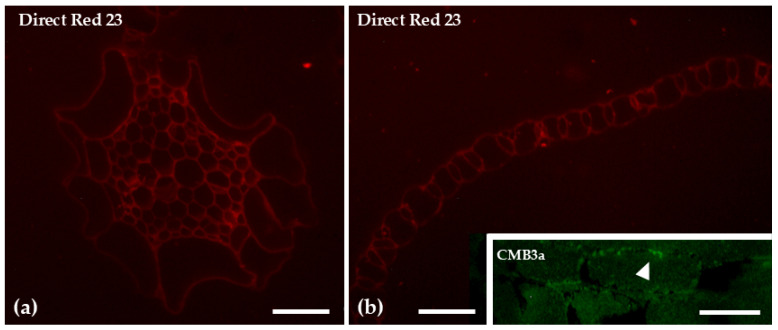
Transverse sections of the gametophyte stem (**a**) and branch leaves (**b**) stained with Direct Red 23 to visualize cellulose, and branch leaves following immunolabeling with the CBM3a antibody to detect crystalline cellulose (inset). Direct Red 23 staining revealed a uniform and evenly distributed signal across the various tissues of both the stem and the leaves. In contrast, the CMB3a antibody, was detected only in the hyaline leaf cells, particularly at their cell wall thickenings (arrowhead in inset). Scale bars: 20 μm.

The branch leaves of *Sphagnum compactum* are unistratose and consist of two distinct cell types: large, water-filled hyaline cells (hyalocysts) that are dead at maturity and often possess pores, and chlorophyllous cells (chlorocysts) arranged around the hyalocysts. In the stem, the epidermis is composed exclusively of hyaline cells (arrow), while in the center (bracket) there is the central cylinder, as observed in toluidine blue-stained cross-sections (Figure 1). Direct Red 23 staining for cellulose revealed a uniform and evenly distributed signal across the various tissues of both the stem and the leaves. In contrast, when CBM3a antibody was used epitopes, specifically used for crystalline cellulose, were detected only in the hyaline leaf cells, particularly at their cell wall thickenings (Figure 2).

**Figure 3:**
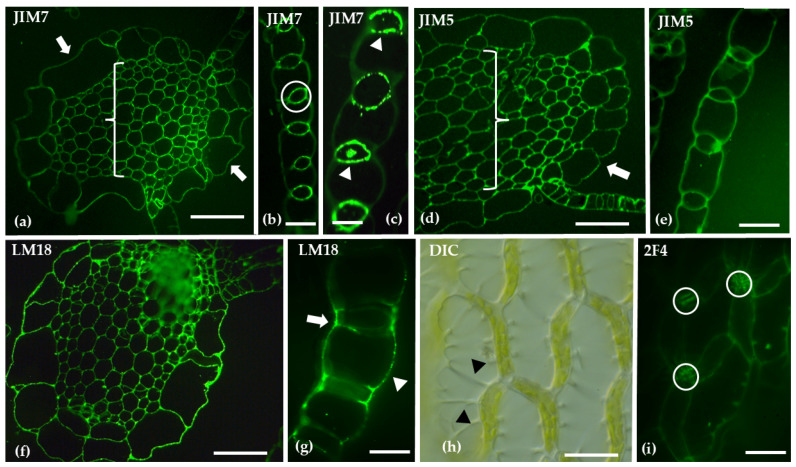
Transverse sections of the gametophyte stem (**a**,**d**,**f**) and branch leaves (**b**,**c**,**e**,**g**), along with surface views of a branch leaf (**h**,**i**), were analyzed following immunolabeling with JIM7 (**a**–**c**), JIM5 (**d**,**e**), LM18 (**f**,**g**), and 2F4 (**i**), or viewed using DIC microscopy (**h**). In the stem, the hyaline cell epidermis showed weak JIM7 labeling (arrows in (**a**)), while the central cylinder (bracket) exhibited stronger labeling (**a**). In the branch leaves, JIM7 was predominantly localized in the photosynthetic cells (circle and arrowheads in **b** and **c**). The hyaline cell epidermis of the stem showed weak JIM5 labeling (arrow in (**d**)), with stronger labeling in the central cylinder (bracket). In the branch leaves, JIM5 labeling was present in both hyaline and photosynthetic cells (**e**). LM18 labeling was widespread in the stem (**f**), while in the branch leaves, strong labeling was observed on the outer walls of both hyaline cells (arrowhead) and photosynthetic cells (arrow in (**g**)). Hyaline cells in the branch leaves of *S. compactum* exhibited spirally arranged thickenings in their cell walls (arrowheads in (**h**)), and the 2F4 antibody was localized on the transverse walls of chlorocyst cells (circles in (**i**)). Scale bars: (**a**,**b**,**d**–**i**) 20 μm; (**c**) 10 μm.

Distinct patterns of homogalacturonan (HG) distribution were revealed through immunolabeling. JIM7, which detects methyl-esterified HGs, exhibited weak labeling in the hyaline cell epidermis of the stem and stronger labeling in the central cylinder. In branch leaves, JIM7 was predominantly localized to the photosynthetic cells. JIM5, specifically used for de-methyl-esterified HGs, demonstrated weak labeling in the hyaline cell epidermis of the stem and stronger labeling in the central cylinder. In branch leaves, JIM5 showed extensive labeling in both hyaline and photosynthetic cells. LM18, recognizing both unmethyl-esterified and de-methyl-esterified HGs, displayed widespread labeling in the stem and showed strong labeling on the outer walls of both hyaline and photosynthetic cells in the branch leaves. Finally, 2F4, which detects partially or non-methyl-esterified HGs cross-linked with calcium bridges, highlighted spirally arranged thickenings in the walls of hyaline cells in the branch leaves, with labeling focused on the transverse walls of chlorocyst cells (Figure 3).

**Figure 4:**
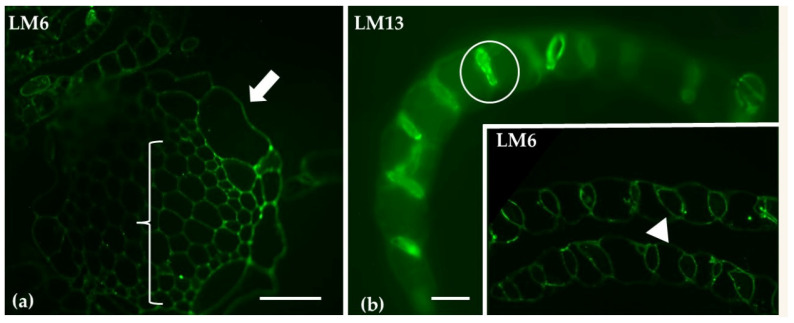
Transverse sections of the gametophyte stem (**a**) and branch leaves (**b**), following immunolabeling with LM6 (**a**; Inset) or LM13 (**b**) to detect arabinan. The epidermis of hyaline cells in the stem (arrows) shows weak labeling, as does the central cylinder (bracket). In the branch leaves, both LM6 and LM13 are predominantly localized in the photosynthetic cells (circle in (**b**) and arrowhead in inset). Scale bars: (**a**) 20 μm; (**b**, inset): 10 μm.

Immunolabeling with LM6 and LM13 antibodies, specific for arabinans, showed pronounced labeling in the cell walls of photosynthetic cells, whereas the stem exhibited a reduced signal (Figure 4).

**Figure 5:**
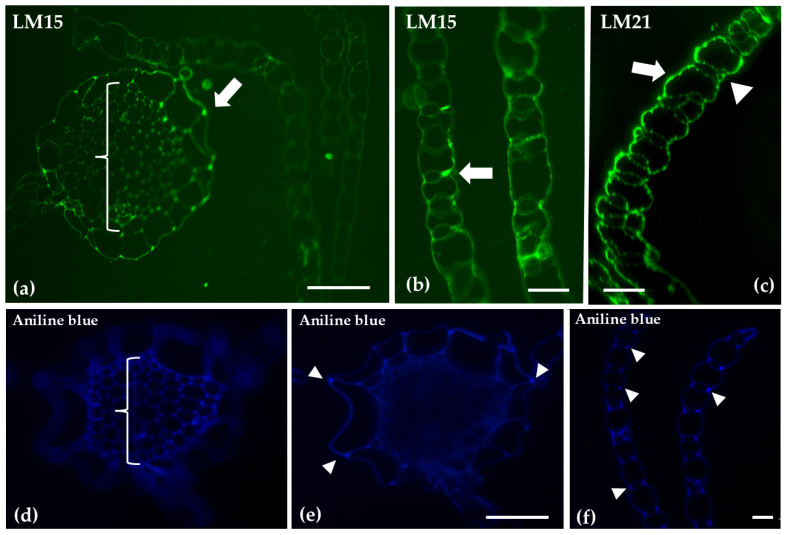
Transverse sections of the gametophyte stem (**a**,**d**,**e**) and branch leaves (**b**,**c**,**f**), following immunolabeling with LM15, LM21 detecting xyloglucans and mannans, or aniline blue for callose staining, respectively. The epidermis of hyaline cells in the stem (arrows) shows LM15 labeling at cell junctions, as does the central cylinder (bracket) (**a**). In the branch leaves, LM15 is primarily localized at the junctions between photosynthetic cells and hyaline cells (arrow) (**b**), while LM21 is present in the outer cell walls of both hyaline cells and photosynthetic cells (arrow and arrowhead, respectively) (**c**). Aniline blue was detected in the central cylinder (bracket, (**d**)), at the cell junctions of the hyaline cells in the stem epidermis (arrowheads, (**e**)), and at the cell junctions of the photosynthetic cells in the leaves (arrowheads, (**f**)). Scale bars: 20 μm.

The distribution of xyloglucans, mannans and callose was assessed using LM15 LM21 antibodies, and aniline blue stain, respectively. Xyloglucans were predominantly localized at the cell junctions in both stem and branch leaves. Mannans were present along the outer cell walls of both hyaline and photosynthetic cells. Aniline blue staining was particularly intense in the central cylinder, as well as at the cell junctions of hyaline stem cells and photosynthetic cells in the leaves (Figure 5).

**Figure 6:**
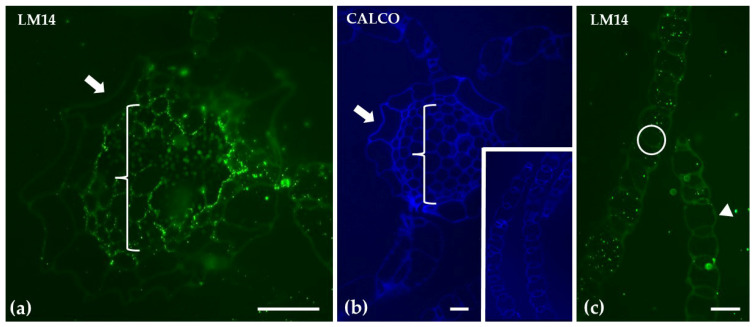
Transverse sections of the gametophyte stem (**a**) and branch leaves (inset in **b**,**c**) were analyzed following immunolabeling with LM14 (**a**,**c**) to detect arabinogalactan proteins, or calcofluor staining (**b**). LM14 labeling was absent in the epidermis of hyaline cells in the stem (indicated by an arrow in (**a**,**b**)) but was present in the central cylinder (bracket in (**a**,**b**)). In the branch leaves, LM14 was not detected in either the photosynthetic cells or the hyaline cells (indicated by the circle and arrowhead, respectively, in (**c**)). However, the leaves exhibited positive calcofluor staining (inset in **b**). Scale bars: (**a**,**c**) 20 μm; (**b**, inset) 10 μm.

Labeling with LM14, which detects arabinogalactan proteins, revealed no signal in the epidermis of the hyaline cells in the gametophyte stem. However, the central cylinder exhibited clear LM14 labeling. In contrast, LM14 was not detected in branch leaves. Structural visualization with calcofluor staining supported these observations (Figure 6).

### 2.2. Marchantia polymorpha

**Figure 7:**
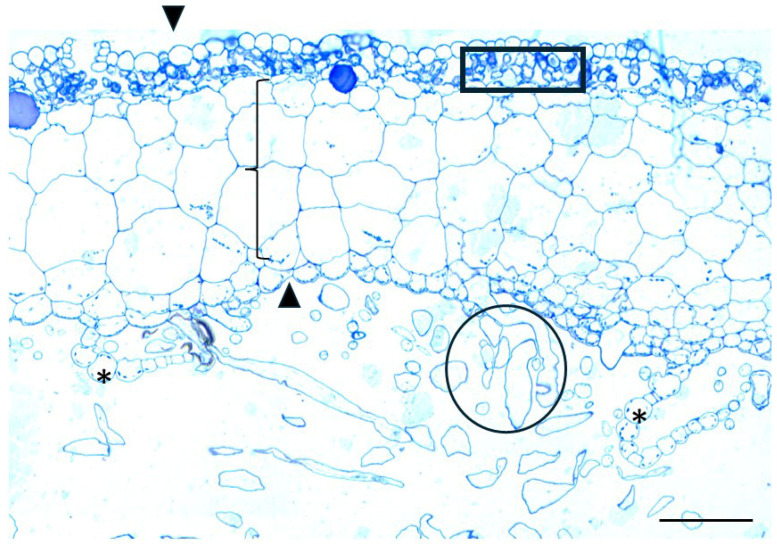
Cross-section of the thallus of the gametophyte *Marchantia polymorpha* stained with toluidine blue. The upper and lower epidermis (arrowheads), the chlorophyllous filaments (rectangle), the parenchyma (bracket) between the epidermal layers, and at the lower part of the thallus, the scales (asterisks) and rhizoids (circle) are visible. Scale bar: 50 μm.

**Figure 8:**
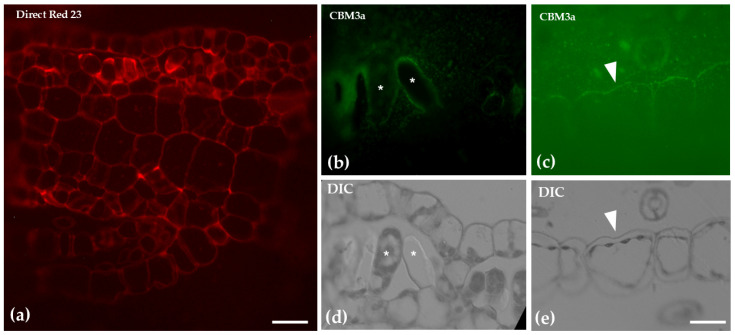
Cross-sections of the thallus stained with Direct Red 23 to visualize cellulose (**a**), after immunolabeling with the CMB3a antibody to detect crystalline cellulose (**b**,**c**) and the sections of **b** and **c**, respectively, with DIC optics (**d**,**e**). Direct Red 23 staining revealed a uniform and evenly distributed signal across the various tissues. CBM3a antibody was detected only in the chlorophyllous filaments (asterisks in (**b**,**d**)) and in the scale cell walls (arrowhead in (**c**,**e**)). Scale bars: 20 μm.

A cross-section of the thallus of the gametophyte *Marchantia polymorpha* stained with toluidine blue reveals a complex, three-layered structure. The upper assimilatory region forms distinct “air chambers”, housing chlorophyllous filaments, which are essential for photosynthesis. The middle storage region consists of parenchyma cells, serving as a reservoir for nutrients. The ventral epidermis, located at the lower part of the thallus, contains scales and rhizoids. The entire structure is enclosed by the upper and lower epidermis (Figure 7). Direct Red 23 staining for cellulose revealed a uniform and evenly distributed signal across the various tissues of the thallus. CMB3a antibody, specific for crystalline cellulose, was detected only in the chlorophyllous filaments and the scale cell walls, particularly at their cell wall thickenings (Figure 8).

**Figure 9:**
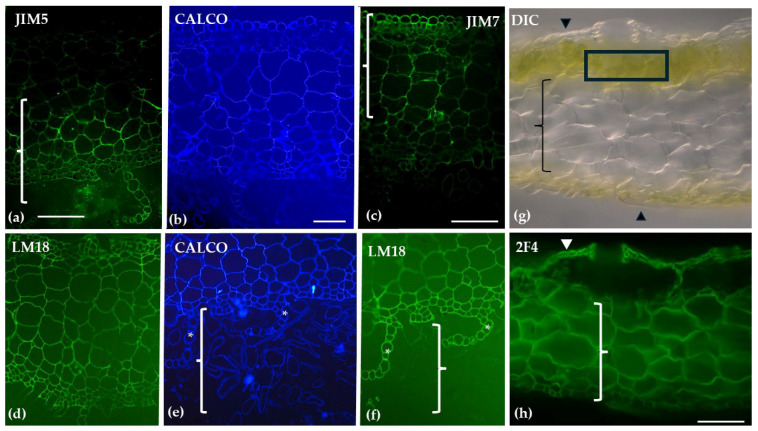
Transverse sections of the gametophyte thallus following immunolabeling with various antibodies and stains. (**a**) JIM5 labeling was observed in the lower parenchyma (bracket) and scales. (**c**) JIM7 labeling was detected in the upper epidermis and upper parenchyma (bracket). (**d**,**f**) LM18 labeling indicated a widespread distribution of homogalacturonans (HGs) across all histological regions, including the scales (asterisks in (**f**)), No labeling was observed in the rhizoids (bracket in (**f**) and corresponding calcofluor staining in (**e**)). (**h**) The 2F4 staining was present in the upper epidermis (arrowhead in (**h**) and the corresponding DIC image in (**g**)) and in the parenchyma (bracket in (**h**) and corresponding DIC in (**g**)), but was absent from the chlorophyllous filaments (rectangle in the DIC image, (**g**)) and rhizoids (arrowhead in the corresponding DIC image, (**g**)). (**b**,**e**) Calcofluor white (CALCO) staining highlighted the cell walls in all regions. (**g**) Differential interference contrast (DIC) microscopy image showing the overall structure of the thallus. Scale bars = 50 μm.

Immunolabeling and staining of *Marchantia polymorpha* thallus cross-sections revealed distinct patterns of homogalacturonan (HG) distribution. Using JIM5 and JIM7 antibodies in conjunction with calcofluor-white staining, a clear polarity in HG localization was observed. Methyl-esterified HGs (JIM5) were predominantly found in the lower parenchyma and the scales. In contrast, non-methyl-esterified HGs (JIM7) were localized to the upper epidermis and the upper parenchyma (brackets). Additional immunolabeling with LM18 showed a broader distribution of HGs across all histological regions, including the scales, while rhizoids lacked a detectable HG signal (Figure 9). The 2F4 staining revealed the presence of non-esterified or de-esterified HGAs that are cross-linked by calcium in the upper epidermis and parenchymatic tissue, while being absent from the chlorophyllous filaments (Figure 9).

**Figure 10:**
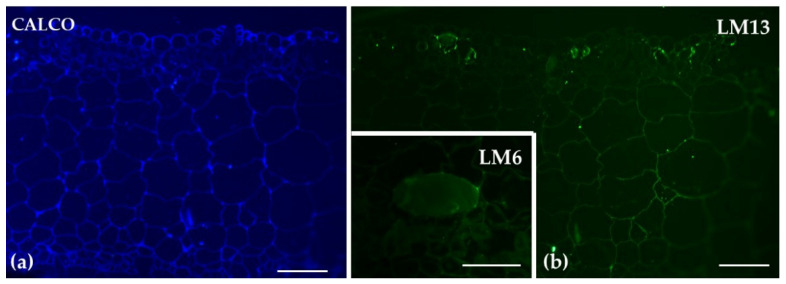
Transverse sections of the gametophyte thallus of *Marchantia polymorpha* analyzed by immunolabeling with LM13 (**b**) and LM6 (inset in (**b**)) as well as with calcofluor white ((**a**) CALCO) staining. LM13 had a low signaling throughout the thallus parenchymatic tissues. LM6 exhibited labelling in the idioblast cells (inset in (**b**)). Scale bars: 50 μm.

Immunolabeling of transverse sections of the gametophyte thallus of *Marchantia polymorpha* with LM13 and LM6, revealed distinct patterns of arabinan distribution. LM13 showed low signaling throughout the parenchyma tissues of the thallus. In contrast, LM6 exhibited labeling specifically in the idioblast cells (Figure 10).

**Figure 11:**
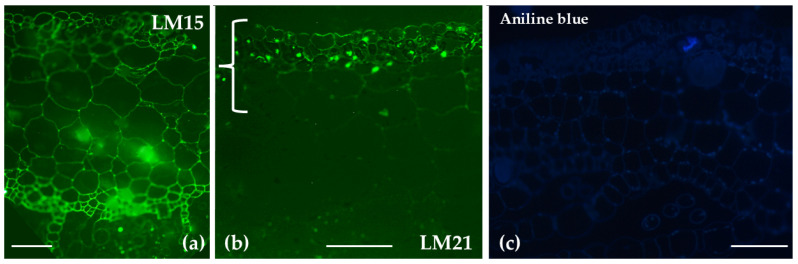
Transverse sections of the gametophyte thallus of *Marchantia polymorpha* analyzed by immunolabeling with LM15 (**a**) and LM21 (**b**) antibodies. LM15 had a broad distribution throughout the thallus. In contrast, LM21 was localized to the upper epidermis, chlorophyllous filaments, and parenchyma cells near the upper epidermis (bracket in (**b**)). No prominent aniline blue signal is detected (**c**). Scale bars: 50 μm.

Transverse sections of the gametophyte thallus of *Marchantia polymorpha* were analyzed following immunolabeling with LM15 and LM21. LM15 labeling, indicating the presence of xyloglucans, showed a broad distribution across the thallus. In contrast, LM21 labeling, specific for mannans, was restricted to the upper epidermis, chlorophyllous filaments, and parenchyma cells located closer to the upper epidermis. No prominent aniline blue signal was detected across the thallus (Figure 11c).

**Figure 12:**
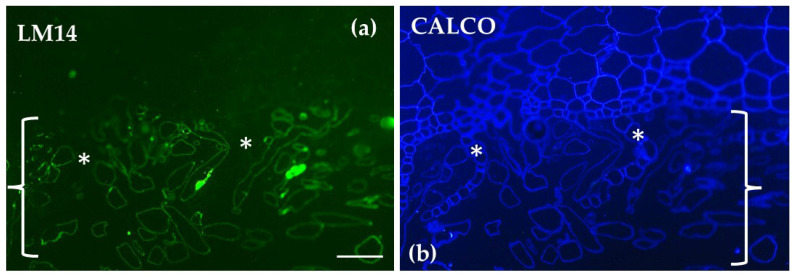
Transverse sections of the gametophyte thallus of *Marchantia polymorpha* following immunolabeling with LM14 (**a**) and staining with calcofluor white (**b**) CALCO. Arabinogalactan proteins (AGPs) are exclusively distributed in the rhizoids (brackets) and are not present in other parts of the thallus, such as the scales (asterisks in (**a**,**b**)). Scale bar: 50 μm.

Immunolabeling with LM14 of transverse sections of the gametophyte thallus of *Marchantia polymorpha* revealed that arabinogalactan proteins (AGPs) are exclusively localized in the rhizoids. No AGP labeling was detected in other parts of the thallus (Figure 12).

### 2.3. Comparison of Labeling Between S. compactum and M. polymorpha

**Figure 13:**
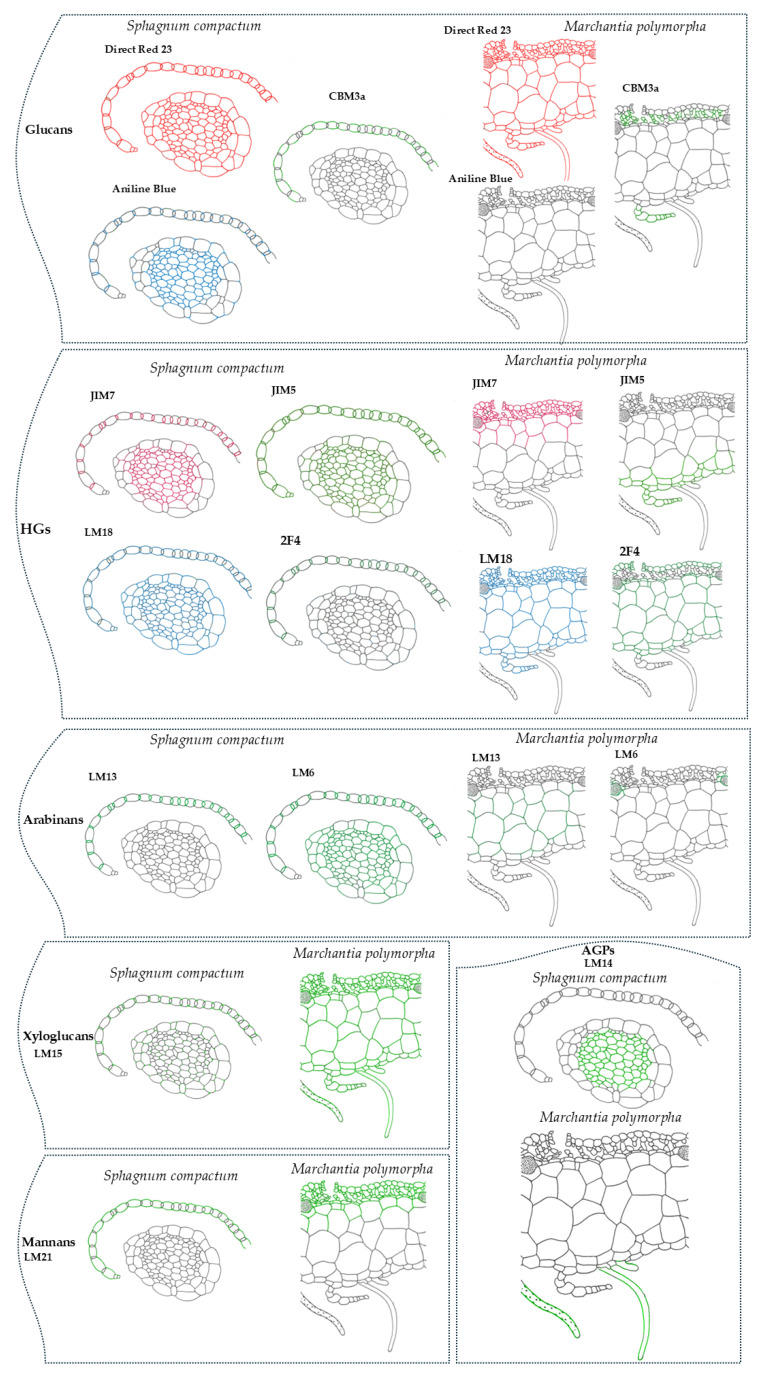
Schematic representation of the data presented in Table 1. Different colors, other than grey, represent the staining or immunolabeling of various detected epitopes or cell wall components as depicted for *S. compactum* and *M. polymorpha*.

Immunolabeling of *S. compactum* and *M. polymorpha* revealed distinct patterns of polysaccharide and protein distribution. In *S. compactum*, homogalacturonans (HGs) were broadly distributed in the stem and branch leaves, with specific labeling in hyaline and photosynthetic cells. Arabinans were predominantly present in photosynthetic cells, while xyloglucans were found at cell junctions and mannans localized to outer cell walls. In *M. polymorpha*, methyl-esterified HGs were mainly in the lower parenchyma and scales, while non-methyl-esterified HGs were localized in the upper epidermis and upper parenchyma. Arabinans were detected in idioblast cells, xyloglucans had a broad distribution, and mannans were restricted to the upper epidermis and chlorophyllous filaments. Arabinogalactan proteins (AGPs) were exclusively found in the rhizoids (Table 1). The schematic representation of the data listed in Table 1 is presented in Figure 13, where different colors—other than grey—indicate the staining or immunolabeling of the various detected epitopes or cell wall components.

## 3. Discussion

The genus *Sphagnum* is unique among mosses in that its leaves are composed of a geometric pattern of interwoven chlorocyst cells that form a network enclosing dead, empty hyaline cells (Figure 3h; [42]). Although hyaline cells are dead and empty at maturity, they remain functional with a primary role in water absorption [43]. Direct Red 23 staining revealed the presence of cellulose throughout *S. compactum* tissues, confirming earlier observations that *Sphagnum* walls are primarily composed of cellulose [36]. When crystalline cellulose epitopes were detected by CBM3a, signaling was scarce, consistent with studies dating back to 1946 that reported similarly low levels in other *Sphagnum* species [44]. Notably, crystalline cellulose was localized within the hyaline cells, suggesting that, due to their water-retaining capacity, these cells may require increased cell wall robustness.

Our results align with previous studies, which indicate a low presence of homogalacturonans (HGs) in the hyaline cells of leaf tissue [36]. However, in our investigation, we clearly observe that the chlorocyst cell walls contain significant levels of methyl-esterified HGs, which likely play a role in optimizing photosynthesis [45]. Additionally, the transverse walls of the chlorocyst cells are rich in HGs linked through calcium bridges, as evidenced by the comparison of Figure 3i with Figure 3h. This finding underscores the importance of cell wall reinforcement, particularly in regions subject to mechanical pressure, such as the chlorocyst cells, which undergo bulging during water storage in the hyaline cells. Calcium-mediated pectin cross-linking, therefore, strengthens cell wall junctions, supporting both water retention and structural integrity [46]. The presence of mannans and xyloglucans further enhances tissue cohesion [47,48], vital for the plant’s overall functionality in wetland ecosystems.

Although our understanding of the occurrence and role of callose across bryophyte taxa and structures remains incomplete, this cell wall polymer is essential for the development and differentiation of bryophyte cells [40]. For instance, in *Physcomitrium* (*Physcomitrella*) *patens*, callose has been localized to the aperture exine in developing spores. The same study [49] identified 12 *P. patens* callose synthase genes, suggesting multiple roles for callose in both development and stress response. In our study, callose staining was prominent at the cell junctions of chlorophyllous and hyaline cells, indicating that these sites may be under mechanical stress, with callose potentially playing a reinforcing role.

The LM6 and LM13 antibodies employed in this study were able to detect arabinans, which are important components of the cell walls in chlorocyst cells. Arabinans are known for their role in maintaining cell wall flexibility and supporting cell expansion. As such, they likely facilitate the dynamic regulation of cell wall porosity and elasticity, both of which are essential for optimizing light capture and improving metabolic efficiency in photosynthetic tissues [50].

Furthermore, the stem of *Sphagnum* is internally divided into the cortex and central cylinder, with the internal stem cells exhibiting a highly specialized cytoplasmic organization, including a well-developed network of endoplasmic microtubules, which relates them to food-conducting cells in other mosses [51]. This specialized cytoplasmic structure may be mirrored in the composition of the cell wall, where the presence of HGs likely plays a role in supporting the transport functions of the central cylinder cells [51].

*M. polymorpha* is widely recognized as a model organism in bryophyte research due to its relatively simple structure that has been studied in detail. As a non-vascular plant, it provides valuable insights into the evolutionary development of plant morphology and cellular functions [28]. Regarding its cell wall composition, previous studies on the cell wall fractions of *M. polymorpha* have demonstrated the presence of key polysaccharides commonly found in flowering plants, such as homogalacturonans (HGs), rhamnogalacturonan-I (RG-I), xyloglucans (XGs), and xylans [52]. Additionally, mannans, which are also found in charophyte algae are present in their cell walls [53]. A common method for labeling cellulose is Calcofluor White, also applied in this study. In numerous investigations of plant cell walls, this dye has shown positive staining in liverwort cell walls (as reviewed in [17]; this study), reinforcing the observation that cellulose is present in all land plants [54]. The use of Direct Red 23 staining further confirmed these findings. Additionally, detection of crystalline cellulose via CBM3a antibody revealed the presence of the specific epitope in low abundance and confined to specific cell types (e.g., chlorophyllous filaments and scales).

In the present study, low-methylesterified homogalacturonans (HGs) are the predominant pectins detected in the cell walls of *M. polymorpha*, consistent with previous findings [31]. Notably, this study presents, for the first time, a distinct polarization in the distribution of methylation states of HGs. Methylesterified HGs are primarily located in the upper epidermis and the upper parenchyma, while demethylesterified HGs are mainly found in the lower epidermis and rhizoids. The photosynthetic parenchyma, however, is devoid of demethylesterified HGs associated with calcium bridges and is instead enriched with methylesterified HGs in the same thallus region. To date, six pectin methylesterases (PMEs) have been identified in the *M. polymorpha* cell wall proteome [32], but no PME inhibitors have been predicted in its genome. As previously noted, [55,56], low-methylesterified HGs can form calcium bridges when demethylation occurs in a block-wise manner. This fine-tuned demethylesterification process can impart different properties to the cell wall, such as increased structural rigidity through calcium-mediated cross-linking or enhanced elasticity when demethylesterification occurs in a non-block-wise manner. The presence of demethylesterified HGs in the lower parenchyma and scales plays a critical role in anchoring and nutrient storage. Furthermore, demethylesterified HGs serve as substrates for enzymes like polygalacturonases or pectate lyases, whose actions result in the release of oligogalacturonides—molecules that are involved in signal transduction [56]. This distribution reflects functional differentiation across tissues [57], with the rigid cell walls in the lower epidermis and rhizoids contributing to anchorage to the substrate.

The presence of mannans in the upper epidermis and chlorophyllous filaments plays a crucial role in tissue stabilization and hydration, helping to maintain water balance and ensuring structural integrity [58,59,60]. In terms of xyloglucans, immunolocalization signals revealed a broad distribution across the thallus, with higher intensity in the lower epidermis and rhizoids, areas where demethylesterified HGs are also present. This observation is in line with previous findings by Kolkas et al. [31], who noted that xyloglucans are more closely associated with a pectin fragment rather than a hemicellulose fragment. This suggests a strong connection between rhamnogalacturonan-I (RG-I) and the side chains of an XXXG motif (X: xylose, G: galactose). Furthermore, glycoside hydrolases of the GH16 family, which include xyloglucan endotransglycosylases/hydrolases (XTHs) [32], have been identified in the genome of *M. polymorpha*, linking their activity to the presence of xyloglucans. This study presents the first report of an association between pectins and xyloglucans in specific *Marchantia* tissues, as demonstrated by an immunolocalization assay. Using aniline blue fluorescence, no callose could be detected, an observation consistent with other studies, such as those on sporophyte seta cells of the leafy liverwort *Lophocolea heterophylla* [61] and in the cell walls of mature elaters and pseudo-elaters of the leafy liverwort *Radula buccinifera* [36].

The presence of tissue-specific components, such as arabinans and glycoproteins, emphasizes the unique characteristics of the cell walls in both *Sphagnum* and *Marchantia*. These components contribute to the specialized structure and function of their cell walls, with arabinans enhancing flexibility [62] and glycoproteins playing a role in structural integrity [63]. Arabinogalactan proteins (AGPs) could play distinct and specialized roles in both *Sphagnum* and *Marchantia*. In *Sphagnum*, AGPs are primarily localized to the central cylinder, the cells of which are thought to support vascular-like functions [51], with AGPs playing a significant role in these processes [64].

Previous studies have identified the polysaccharidic nature of *Marchantia* rhizoid cell walls and noted also the limited presence of callose [65]. Our findings further confirm the presence of AGPs in rhizoids where they may contribute to the anchoring role of the rhizoids. Moreover, AGPs may aid in water retention. In bryophytes, AGPs are enriched with rhamnose and methylated rhamnose at their periphery, creating a less polar surface that could promote hydrophobic interactions, thereby influencing cell wall properties under water stress [15]. The cavitation-resistant and elastic walls of pegged rhizoids, which maintain structural integrity during desiccation [66], could benefit from the presence of AGPs which may also provide water retention capacity. Given the parallelism between the rhizoids of *M. polymorpha* and the water-conducting cells of angiosperms [67], further studies are needed to investigate the structure of the rhizoid cell wall, particularly during its development.

Interestingly, AGPs in both species are highly localized and occur in relatively low abundance, as indicated by previous studies [68,69]. This suggests that their structure may differ from the AGPs typically found in angiosperms, highlighting the unique adaptations of these bryophytes.

Although both bryophytes and vascular plants share fundamental polysaccharides such as cellulose, hemicelluloses, and pectins, bryophytes exhibit unique structural and compositional variations in these components that reflect their distinct evolutionary trajectories. Monosaccharide-linkage analyses have confirmed that although common vascular plant polysaccharides—such as cellulose, xyloglucans, mannans, xylans, and rhamnogalacturonan-type pectins—are present in bryophytes, they possess distinct side chain compositions and configurations (e.g., [6,15,17]). Our complementary immunolabeling, further reveal differential labeling patterns with anti-pectin and anti-arabinogalactan-protein antibodies, emphasizing tissue-specific roles and functional adaptations. The contrasting strategies of *Sphagnum* and *Marchantia* illustrate this divergence: *Sphagnum* thrives in peatlands through specialized hyaline cells that regulate water, store carbon, and shape its habitat, whereas *Marchantia* modifies its cell wall components to balance flexibility, hydration, and structural integrity in variable terrestrial environments [70].

Recent analyses of plant and algal genomes have underscored that modern bryophytes are not primitive relics but rather highly derived lineages. Bryophytes and tracheophytes have experienced similar extensive gene gain and loss relative to the inferred plant ancestral genome, indicating that neither group preserves an ancestral state. In fact, the ancestral angiosperm may have shared more genes with the ancestral land plant than the ancestral liverwort did [71]. Moreover, investigations into cell wall evolution reveal that bryophytes have maintained a mosaic of features—such as cellulose synthase complexes and evolved novel modifications in pectic and hemicellulosic polysaccharides in response to terrestrial stresses [17]. Our findings further indicate that specific tissues, such as the rhizoids of *Marchantia*, where arabinogalactan-protein epitopes are uniquely labeled, highlight the functional diversification within bryophyte cell walls. Τhese insights conjointly challenge the view of bryophytes as simple models for early land plants. The selection of model organisms for studying early plant evolution must be tailored to the trait of interest, integrating a comprehensive appraisal of phylogenetic diversity that includes algal outgroups.

## 4. Materials and Methods

Plant material for the analysis of polysaccharide distribution in cell walls was obtained from axenic cultures of bryophytes grown on solid Gamborg B5 medium supplemented with 1% (*w*/*v*) glucose. Cultures were maintained under controlled growth chamber conditions (temperature: 25 °C, light intensity: 50 μmol m^−2^ s^−1^, photoperiod: 8 h light/16 h dark) [72,73]. The liverwort *Marchantia polymorpha* L. TAK2 (courtesy of Dr. Claus Schwechheimer, Technical University of Munich, Germany) and the moss *Sphagnum compactum* Lam. & DC. were used.

Segments of *S. compactum* gametophytes were fixed in 4% (*w*/*v*) paraformaldehyde (PFA) in PEM buffer (50 mM PIPES, 2 mM EGTA, 2 mM MgSO_4_, pH 6.9) for 1 h. After three washes with PEM, the samples were digested with 2% (*w*/*v*) cellulase (Onozuka R-10, Duchefa Biochemie, Haarlem, The Netherlands) in PEM for 1 h, followed by additional washes and rehydration in extraction solution for 1 h. Samples were then washed in PBS and incubated in T/Ca/S buffer (20 mM Tris-HCl pH 8.2, 0.5 mM CaCl_2_, 150 mM NaCl) before immunolabeling. For the detection of partially or non-methylesterified homogalacturonans crosslinked by calcium bridges, the monoclonal antibody 2F4 (Plant Probes, Leeds, UK) was used. Samples were incubated with 2F4 diluted 1:40 in T/Ca/S for 12 h, washed with the same buffer, and incubated with FITC-conjugated anti-mouse secondary antibody (1:40 dilution) at 37 °C for 2 h, or at room temperature for 12 h. Sections were mounted in an anti-fade solution (2:1 glycerol/PBS with 0.5% p-phenylenediamine). For structural analysis, gametophyte sections were embedded in London Resin White (LRW, Polysciences, Warrington, PA, USA) using mild processing conditions to preserve antigenicity. Semi-thin sections (0.5–2 µm) were prepared using an Ultratome III microtome (LKB-Produkter AB, Stockholm, Sweden) with glass knives [74]. Sections were stained with 0.5% (*w*/*v*) toluidine blue O in 1% (*w*/*v*) borax for general visualization or with 0.05% (*w*/*v*) Calcofluor White (Merck, Darmstadt, Germany) or 0.1% (*w*/*v*) Direct Red 23 (Merck, Darmstadt, Germany) in PBS for cellulose detection [75]. For callose detection, sections were stained with 0.05% (*w*/*v*) aniline blue (Merck, Darmstadt, Germany) in 0.07 M K_2_HPO_4_ buffer (pH 8.5) [76].

Immunolocalization of cell wall epitopes was performed on sections of embedded samples mounted on glass slides (except for the epitope recognized by the 2F4 antibody, as described earlier). Monoclonal antibodies specific to different epitopes (refer to Table 2 for a detailed antibody list) were purchased from Biosupplies Australia (Parkville, Australia) and Plant Probes (Leeds, UK). Initially, blocking was performed by incubating sections in 5% (*w*/*v*) BSA in PBS for 2–3 h, followed by PBS washes and incubation with the primary antibody diluted 1:40 in blocking buffer for at least 12 h. After further washing, sections were incubated with FITC-conjugated secondary antibodies (anti-rat or anti-mouse), diluted 1:40 in blocking buffer, at 37 °C for 2 h or at room temperature for at least 12 h. After a final PBS wash, the sections were mounted with anti-fade mounting medium [77,78,79,80]. Crystalline cellulose was labeled using a His-tagged CBM3 module, detected by a mouse anti-His (Cell Signaling, Danvers, MA, USA) antibody and a FITC-conjugated anti-mouse IgG (Merck, Darmstadt, Germany) [81].

The prepared sections were observed using both bright-field and fluorescence microscopy, as appropriate. Fluorescence microscopy utilized an epifluorescence system equipped with filters specific to each fluorophore, while bright-field observations employed a Zeiss Axioplan microscope (Carl Zeiss AG, Oberkochen, Germany) with a differential interference contrast (DIC) system and an AxioCam MRc5 digital camera. Images were acquired and processed using ZEN 2.0 software. Additionally, all samples were examined for UV autofluorescence (Supplementary Figures S1 and S2). The reliability of the immunolocalization methods was assessed using epifluorescence microscopy, applying the same protocol as in all experiments but omitting the primary antibody and only applying the FITC-conjugated anti-rat antibody. As shown in Supplementary Figures S1 and S2, *S. compactum* and *M. polymorpha* sections do not exhibit any fluorescent signal.

## 5. Conclusions

Our findings highlight the distinct yet functionally significant adaptations in the cell walls of *Sphagnum compactum* and *Marchantia polymorpha*. The differential distribution of polysaccharides such as homogalacturonans, xyloglucans, and mannans, along with the localized presence of AGPs, callose and crystalline cellulose underscores the tissue-specific functional roles of these components in both species. These variations not only contribute to their survival in distinct habitats but also offer insights into the broader evolutionary trajectory of bryophyte cell wall specialization. The unique cell wall structures in these species reflect the close relationship between cellular specialization and environmental adaptation [82]. Further investigation into these cell wall adaptations could provide valuable insights into the evolutionary pathways of bryophytes and their ability to adapt to various ecological niches.

## Figures and Tables

**Table 1:** Summary of immunolabeling results for *Sphagnum compactum* and *Marchantia polymorpha*.

Polysaccharide/ Protein	Antibody/ Label	Distribution in *Sphagnum compactum*	Distribution in *Marchantia polymorpha*
**Glucans**	**Direct Red 23**	Uniform and evenly distributed signal across the various tissues	Uniform and evenly distributed signal across the various tissues
	**CBM3a**	Weak labeling in hyaline leaf cells	Weak labeling in chlorophyllous filaments and scales
	**Aniline Blue**	Staining was observed in the central cylinder of the stem, as well as at the cell junctions of the hyaline stem cells and the photosynthetic cells in the leaves	No prominent signal was detected
**Homogalacturonans (HGs)**	**JIM7**	Weak labeling in hyaline cell epidermis of stem; stronger in central cylinder	Polar distribution to upper epidermis and upper thallus parenchyma
	**LM18**	Widespread in stem; outer walls of hyaline and photosynthetic cells in branch leaves	Broader distribution across all histological regions, including scales; absent in the rhizoids
	**JIM5**	Weak in hyaline cell epidermis of stem; stronger in central cylinder	Polar distribution to the lower epidermis/thallus parenchyma and the scales.
	**2F4**	Labeling was primarily concentrated on the transverse walls of chlorocyst cells in branch leaves	Signaling present in the upper epidermis and parenchymatic tissue, absent from the chlorophyllous filaments
**Arabinans**	**LM6**	Reduced signal in stem; pronounced labeling in photosynthetic cells	Low signal in thallus parenchyma; labeling in idioblast cells
	**LM13**	Pronounced labeling in photosynthetic cells; reduced signal in stem	Low signaling throughout thallus parenchyma
**Xyloglucans**	**LM15**	Predominantly present at cell junctions in stem and branch leaves	Broad distribution across the thallus
**Mannans**	**LM21**	Found in outer cell walls of hyaline and photosynthetic cells	Restricted to the upper epidermis, chlorophyllous filaments, and parenchyma closer to the upper epidermis
**Arabinogalactan Proteins (AGPs)**	**LM14**	No signal in epidermis of hyaline cells; clear signal in central cylinder	Exclusively localized in the rhizoids; absent in other parts of the thallus

**Table 2:** List of primary monoclonal antibodies used in the current study for immunolocalization of different cell wall epitopes [77,78,79,80,81]. All descriptions and/or references for the antibodies and their use can be found at www.kerafast.com (accessed on 5 February 2025) and www.plantprobes.net (accessed on 5 February 2025).

Antibody	Epitope Recognized
	*Crystalline cellulose*
CBM3a	Carbohydrate Binding Module family 3
	*Hemicelluloses*
LM15	XXLG and XLLG motifs of xyloglucan
LM21	β-(1-4)-Manno-oligosaccharides from DP2 to DP5
	*Pectins*
LM6	(1→5)-α-L-arabinans
LM18	Homogalacturonan (HG) domain of pectic polysaccharides (binds to both partially methyl-esterified and un-esterified HGs)
JΙΜ5	HG domain of pectic polysaccharides (binds strongly to un-esterified HGs)
JIM7	HG domain of pectic polysaccharides (requires methyl-esters for HG recognition, does not bind to un-esterified HGs)
2F4	Non-esterified or de-esterified HGAs that are cross-linked by calcium
	*Arabinogalactan Proteins* (*AGPs*)
LM14	Arabinogalactan/AGP

## Data Availability

Data will be available upon request.

## Supplementary Materials

The supporting information can be downloaded at: https://www.mdpi.com/article/10.3390/ijms26083602/s1.

## References

[1] Sørensen I., Domozych D., Willats W.G. (2010). How Have Plant Cell Walls Evolved?. Plant Physiol..

[2] Sarkar P., Bosneaga E., Auer M. (2009). Plant Cell Walls Throughout Evolution: Towards a Molecular Understanding of Their Design Principles. J. Exp. Bot..

[3] Plancot B., Gügi B., Mollet J.C., Loutelier-Bourhis C., Giovind S.R., Lerouge P., Follet-Gueye M.L., Vicré M., Alfonso C., Nguema-Ona E. (2019). Dessication tolerance in plants: Structural charactérization of the cell wall hemicellulosic polysaccharides in three Selaginella species. Carbohydr. Polym..

[4] Soriano G., Del-Castillo-Alonso M.A., Monforte L., Núñez-Olivera E., Martínez-Abaigar J. (2019). Phenolic compounds from different bryophyte species and cell compartments respond specifically to ultraviolet radiation, but not particularly quickly. Plant Physiol. Biochem..

[5] Niklas K.J., Cobb E.D., Matas A.J. (2017). The evolution of hydrophobic cell wall biopolymers: From algae to angiosperms. J. Exp. Bot..

[6] Fuertes-Rabanal M., Rebaque D., Largo-Gosens A., Encina A., Mélida H. (2024). Cell Walls, a Comparative View of the Composition of Cell Surfaces of Plants, Algae and Microorganisms. J. Exp. Bot..

[7] Shaw A.J., Szövényi P., Shaw B. (2011). Bryophyte Diversity and Evolution: Windows into the Early Evolution of Land Plants. Am. J. Bot..

[8] Ogwu M.C. (2020). Ecological and Economic Significance of Bryophytes. Current State and Future Impacts of Climate Change on Biodiversity.

[9] Vitt D.H., House M. (2021). Bryophytes as Key Indicators of Ecosystem Function and Structure of Northern Peatlands. Bryophyt. Divers. Evol..

[10] Chen K.H., Nelson J. (2022). A Scoping Review of Bryophyte Microbiota: Diverse Microbial Communities in Small Plant Packages. J. Exp. Bot..

[11] Yadav S., Basu S., Srivastava A., Biswas S., Mondal R., Jha V.K., Mishra Y. (2023). Bryophytes as Modern Model Plants: An Overview of Their Development, Contributions, and Future Prospects. J. Plant Growth Regul..

[12] Flores-Sandoval E., Eklund D.M., Bowman J.L. (2015). A Simple Auxin Transcriptional Response System Regulates Multiple Morphogenetic Processes in the Liverwort *Marchantia polymorpha*. PLoS Genet..

[13] Ligrone R., Duckett J.G., Renzaglia K.S. (2012). Major Transitions in the Evolution of Early Land Plants: A Bryological Perspective. Ann. Bot..

[14] Slate M.L., Antoninka A., Bailey L., Berdugo M.B., Callaghan D.A., Cárdenas M., Coe K.K. (2024). Impact of Changing Climate on Bryophyte Contributions to Terrestrial Water, Carbon, and Nitrogen Cycles. New Phytol..

[15] Pfeifer L., Mueller K.-K., Classen B. (2022). The cell wall of hornworts and liverworts: Innovations in early land plant evolution?. J. Exp. Bot..

[16] Roig-Oliver M., Douthe C., Bota J., Flexas J. (2021). Cell Wall Thickness and Composition Are Related to Photosynthesis in Antarctic Mosses. Physiol. Plant..

[17] Popper Z.A., Fry S.C. (2003). Primary cell wall composition of bryophytes and charophytes. Ann. Bot..

[18] Peña M.J., Darvill A.G., Eberhard S., York W.S., O’Neill M.A. (2008). Moss and liverwort xyloglucans contain galacturonic acid and are structurally distinct from the xyloglucans synthesized by hornworts and vascular plants. Glycobiology.

[19] Medina R., Johnson M.G., Liu Y., Wickett N.J., Shaw A.J., Goffinet B. (2019). Phylogenomic Delineation of *Physcomitrium* (Bryophyta: Funarineae) Based on Targeted Sequencing of Nuclear Exons and Their Flanking Regions Rejects the Retention of *Physcomitrella*, *Physcomitridium*, and *Aphanorrhegma*. J. Syst. Evol..

[20] Shaw A.J., Schmutz J., Devos N., Shu S., Carrell A.A., Weston D.J. (2016). The Sphagnum Genome Project: A New Model for Ecological and Evolutionary Genomics. Adv. Bot. Res..

[21] Shaw J.A., Devos N., Liu Y., Cox C.J., Goffinet B., Flatberg K.I., Shaw B. (2016). Organellar Phylogenomics of an Emerging Model System: Sphagnum (Peatmoss). Ann. Bot..

[22] Klavina L., Ramawat K., Mérillon J.M. (2015). Polysaccharides from Lower Plants: Bryophytes. Polysaccharides.

[23] Hájek T., Beckett R.P. (2008). Effect of water content components on desiccation and recovery in *Sphagnum* mosses. Ann. Bot..

[24] Ballance S., Kristiansen K.A., Skogaker N.T., Tvedt K.E., Christensen B.E. (2012). The Localisation of Pectin in Sphagnum Moss Leaves and Its Role in Preservation. Carbohydr. Polym..

[25] Hymas M., Casademont-Reig I., Poigny S., Stavros V.G. (2023). Characteristic Photoprotective Molecules from the Sphagnum World: A Solution-Phase Ultrafast Study of Sphagnic Acid. Molecules.

[26] Bryan L., Shaw R., Schoonover E., Koehl A., DeVries-Zimmerman S., Philben M. (2024). Sphagnan in Sphagnum-Dominated Peatlands: Bioavailability and Effects on Organic Matter Stabilization. Biogeochemistry.

[27] Tveit A.T., Kiss A., Winkel M., Horn F., Hájek T., Svenning M.M., Liebner S. (2020). Environmental Patterns of Brown Moss- and Sphagnum-Associated Microbial Communities. Sci. Rep..

[28] Bowman J.L., Arteaga-Vazquez M., Berger F., Briginshaw L.N., Carella P., Aguilar-Cruz A., Zachgo S. (2022). The Renaissance and Enlightenment of Marchantia as a Model System. Plant Cell.

[29] Shimamura M. (2016). *Marchantia polymorpha*: Taxonomy, Phylogeny and Morphology of a Model System. Plant Cell Physiol..

[30] Bowman J.L. (2022). The Liverwort *Marchantia polymorpha*, a Model for All Ages. Curr. Top. Dev. Biol..

[31] Kolkas H., Burlat V., Jamet E. (2023). Immunochemical identification of the main cell wall polysaccharides of the early land plant *Marchantia polymorpha*. Cells.

[32] Kolkas H., Balliau T., Chourré J., Zivy M., Canut H., Jamet E. (2022). The Cell Wall Proteome of Marchantia polymorpha Reveals Specificities Compared to Those of Flowering Plants. Front. Plant Sci..

[33] Apostolakos P., Galatis B. (1985). Studies on the Development of the Air Pores and Air Chambers of Marchantia paleacea: III. Microtubule Organization in Preprophase-Prophase Initial Aperture Cells—Formation of Incomplete Preprophase Microtubule Bands. Protoplasma.

[34] Jibran R., Hill S.J., Lampugnani E.R., Hao P., Doblin M.S., Bacic A., Brummell D.A. (2024). The Auronidin Flavonoid Pigments of the Liverwort Marchantia polymorpha Form Polymers That Modify Cell Wall Properties. Plant J..

[35] Popper Z.A. (2008). Evolution and diversity of green plant cell walls. Curr. Opin. Plant Biol..

[36] Kremer C., Pettolino F., Bacic A., Drinnan A. (2004). Distribution of cell wall components in Sphagnum hyaline cells and in liverwort and hornwort elaters. Planta.

[37] Carafa A., Duckett J.G., Knox J.P., Ligrone R. (2005). Distribution of cell-wall xylans in bryophytes and tracheophytes: New insights into basal interrelationships of land plants. New Phytol..

[38] Wallace S., Fleming A., Wellman C.H., Beerling D.J. (2011). Evolutionary Development of the Plant and Spore Wall. AoB Plants.

[39] Henry J.S., Lopez R.A., Renzaglia K.S. (2020). Differential Localization of Cell Wall Polymers across Generations in the Placenta of *Marchantia polymorpha*. J. Plant Res..

[40] Renzaglia K., Duran E., Sagwan-Barkdoll L., Henry J. (2024). Callose in Leptoid Cell Walls of the Moss *Polytrichum* and the Evolution of Callose Synthase across Bryophytes. Front. Plant Sci..

[41] Ligrone R., Vaughn K.C., Renzaglia K.S., Knox J.P., Duckett J.G. (2002). Diversity in the Distribution of Polysaccharide and Glycoprotein Epitopes in the Cell Walls of Bryophytes: New Evidence for the Multiple Evolution of Water-Conducting Cells. New Phytol..

[42] Anderson L.E., Ammann K. (1991). Cell Wall Ornamentation in the Hyaline Cells of *Sphagnum*. J. Hattori Bot. Lab..

[43] Kremer C.L., Drinnan A.N. (2004). Secondary Walls in Hyaline Cells of *Sphagnum*. Aust. J. Bot..

[44] Plank N. (1946). The Nature of Cellulose in *Sphagnum*. Am. J. Bot..

[45] Weraduwage S.M., Kim S.J., Renna L., Anozie F.C., Sharkey T.D., Brandizzi F. (2016). Pectin methylesterification impacts the relationship between photosynthesis and plant growth. Plant Physiol..

[46] Daher F.B., Braybrook S.A. (2015). How to Let Go: Pectin and Plant Cell Adhesion. Front. Plant Sci..

[47] Fradera-Soler M., Grace O.M., Jørgensen B., Mravec J. (2022). Elastic and collapsible: Current understanding of cell walls in succulent plants. J. Exp. Bot..

[48] Ropitaux M., Bernard S., Follet-Gueye M.L., Vicré M., Boulogne I., Driouich A. (2019). Xyloglucan and cellulose form molecular cross-bridges connecting root border cells in pea (*Pisum sativum*). Plant Physiol. Biochem..

[49] Schuette S., Wood A.J., Geisler M., Geisler-Lee J., Ligrone R., Renzaglia K.S. (2009). Novel Localization of Callose in the Spores of *Physcomitrella patens* and Phylogenomics of the Callose Synthase Gene Family. Ann. Bot..

[50] Verhertbruggen Y., Marcus S.E., Haeger A., Verhoef R., Schols H.A., McCleary B.V., Knox J.P. (2009). Developmental Complexity of Arabinan Polysaccharides and Their Processing in Plant Cell Walls. Plant J..

[51] Ligrone R., Duckett J.G. (1998). The leafy stems of Sphagnum (Bryophyta) contain highly differentiated polarized cells with axial arrays of endoplasmic microtubules. New Phytol..

[52] Popper Z.A., Michel G., Hervé C., Domozych D.S., Willats W.G., Tuohy M.G., Stengel D.B. (2011). Evolution and Diversity of Plant Cell Walls: From Algae to Flowering Plants. Annu. Rev. Plant Biol..

[53] Melton L.D., Smith B.G., Ibrahim R., Schröder R. (2009). Mannans in Primary and Secondary Plant Cell Walls. N. Z. J. For. Sci..

[54] Popper Z.A., Tuohy M.G. (2010). Beyond the green: Understanding the evolutionary puzzle of plant and algal cell walls. Plant Physiol..

[55] Giannoutsou E., Apostolakos P., Galatis B. (2016). Spatio-temporal Diversification of the Cell Wall Matrix Materials in the Developing Stomatal Complexes of *Zea mays*. Planta.

[56] Giannoutsou E., Sotiriou P., Nikolakopoulou T.L., Galatis B., Apostolakos P. (2020). Callose and Homogalacturonan Epitope Distribution in Stomatal Complexes of *Zea mays* and *Vigna sinensis*. Protoplasma.

[57] Apostolakos P., Livanos P., Giannoutsou E., Panteris E., Galatis B. (2018). The Intracellular and Intercellular Cross-Talk during Subsidiary Cell Formation in *Zea mays*: Existing and Novel Components Orchestrating Cell Polarization and Asymmetric Division. Ann. Bot..

[58] Kohchi T., Yamato K.T., Ishizaki K., Yamaoka S., Nishihama R. (2021). Development and molecular genetics of *Marchantia polymorpha*. Annu. Rev. Plant Biol..

[59] Rodríguez-Gacio M.D.C., Iglesias-Fernández R., Carbonero P., Matilla Á.J. (2012). Softening-up mannan-rich cell walls. J. Exp. Bot..

[60] Brett C.T., Baydoun E.H., Abdel-Massih R.M. (2005). Pectin-xyloglucan linkages in type I primary cell walls of plants. Plant Biosyst..

[61] Thomas R.J. (1977). Wall Analyses of *Lophocolea seta* Cells (Bryophyta) Before and After Elongation. Plant Physiol..

[62] Carroll S., Amsbury S., Durney C.H., Smith R.S., Morris R.J., Gray J.E., Fleming A.J. (2022). Altering Arabinans Increases *Arabidopsis* Guard Cell Flexibility and Stomatal Opening. Curr. Biol..

[63] Ellis M., Egelund J., Schultz C.J., Bacic A. (2010). Arabinogalactan-Proteins: Key Regulators at the Cell Surface?. Plant Physiol..

[64] Casero P.J., Casimiro I., Knox J.P. (1998). Occurrence of Cell Surface Arabinogalactan-Protein and Extensin Epitopes in Relation to Pericycle and Vascular Tissue Development in the Root Apex of Four Species. Planta.

[65] Cao J.G., Dai X.L., Zou H.M., Wang Q.X. (2014). Formation and development of rhizoids of the liverwort *Marchantia polymorpha*. J. Torrey Bot. Soc..

[66] Duckett J.G., Ligrone R., Renzaglia K.S., Pressel S. (2014). Pegged and smooth rhizoids in complex thalloid liverworts (Marchantiopsida): Structure, function and evolution. Bot. J. Linn. Soc..

[67] Lu Y.T., Loue-Manifel J., Bollier N., Gadient P., De Winter F., Carella P., Goodrich J. (2024). Convergent evolution of water-conducting cells in *Marchantia* recruited the ZHOUPI gene promoting cell wall reinforcement and programmed cell death. Curr. Biol..

[68] Happ K., Classen B. (2019). Arabinogalactan-proteins from the liverwort *Marchantia polymorpha* L., a member of a basal land plant lineage, are structurally different to those of angiosperms. Plants.

[69] Bartels D., Baumann A., Maeder M., Geske T., Heise E.M., von Schwartzenberg K., Classen B. (2017). Evolution of plant cell wall: Arabinogalactan-proteins from three moss genera show structural differences compared to seed plants. Carbohydr. Polym..

[70] Stech M., Câmara P.E., Medina R., Muñoz J. (2021). Advances and challenges in bryophyte biology after 50 years of International Association of Bryologists. Bryophyt. Divers. Evol..

[71] Harris B.J., Clark J.W., Schrempf D., Szöllősi G.J., Donoghue P.C., Hetherington A.M., Williams T.A. (2022). Divergent Evolutionary Trajectories of Bryophytes and Tracheophytes from a Complex Common Ancestor of Land Plants. Nat. Ecol. Evol..

[72] González M.L., Mallon R., Reinoso J., Rodríguez-Oubina J. (2006). In vitro micropropagation and long-term conservation of the endangered moss *Splachnum ampullaceum*. Biol. Plant..

[73] Nelson J.M. (2017). Diversity and Effects of the Fungal Endophytes of the Liverwort *Marchantia polymorpha*. Ph.D. Dissertation.

[74] Meidani C., Ntalli N.G., Giannoutsou E., Adamakis I.D.S. (2019). Cell wall modifications in giant cells induced by the plant parasitic nematode *Meloidogyne incognita* in wild-type (Col-0) and the fra2 *Arabidopsis thaliana* katanin mutant. Int. J. Mol. Sci..

[75] Ursache R., Andersen T.G., Marhavý P., Geldner N. (2018). A Protocol for Combining Fluorescent Proteins with Histological Stains for Diverse Cell Wall Components. Plant J..

[76] Smith M.M., McCully M.E. (1978). A Critical Evaluation of the Specificity of Aniline Blue Induced Fluorescence. Protoplasma.

[77] Giannoutsou E., Galatis B., Apostolakos P. (2019). De-esterified homogalacturonan enrichment of the cell wall region adjoining the preprophase cortical cytoplasmic zone in some protodermal cell types of three land plants. Int. J. Mol. Sci..

[78] Sotiriou P., Giannoutsou E., Panteris E., Galatis B., Apostolakos P. (2018). Local differentiation of cell wall matrix polysaccharides in sinuous pavement cells: Its possible involvement in the flexibility of cell shape. Plant Biol..

[79] Pappas D., Giannoutsou E., Panteris E., Gkelis S., Adamakis I.D.S. (2022). Microcystin-LR and cyanobacterial extracts alter the distribution of cell wall matrix components in rice root cells. Plant Physiol. Biochem..

[80] Azariadis A., Vouligeas F., Salame E., Kouhen M., Rizou M., Blazakis K., Sotiriou P., Ezzat L., Mekkaoui K., Monzer A. (2023). Response of Prolyl 4 Hydroxylases, Arabinogalactan Proteins and Homogalacturonans in Four Olive Cultivars under Long-Term Salinity Stress in Relation to Physiological and Morphological Changes. Cells.

[81] Gkolemis K., Giannoutsou E., Adamakis I.S., Galatis B., Apostolakos P. (2023). Cell wall anisotropy plays a key role in *Zea mays* stomatal complex movement: The possible role of the cell wall matrix. Plant Mol. Biol..

[82] He X., He K.S., Hyvönen J. (2016). Will Bryophytes Survive in a Warming World?. Perspect. Plant Ecol. Evol. Syst..

