# Supplementary material for: Tissue-Specific Differential Distribution of Cell Wall Epitopes in Sphagnum compactum and Marchantia polymorpha
Source: Int J Mol Sci. 2025 Apr 11;26(8):3602. doi: 10.3390/ijms26083602 (PMC12026656; doi:10.3390/ijms26083602)
Supplement: Supplementary file 1 [file ijms-26-03602-s001.zip › ijms-3506020-supplementary.pdf]

Tissue-Specific Differential Distribution of Cell Wall Epitopes in *Sphagnum compactum* and *Marchantia polymorpha*

Ioannis-Dimosthenis S. Adamakis [1,*], Penelope Sotiriou [1], Natalia Ntanou [1], Jessica M. Nelson [2] and Eleni Giannoutsou [1]

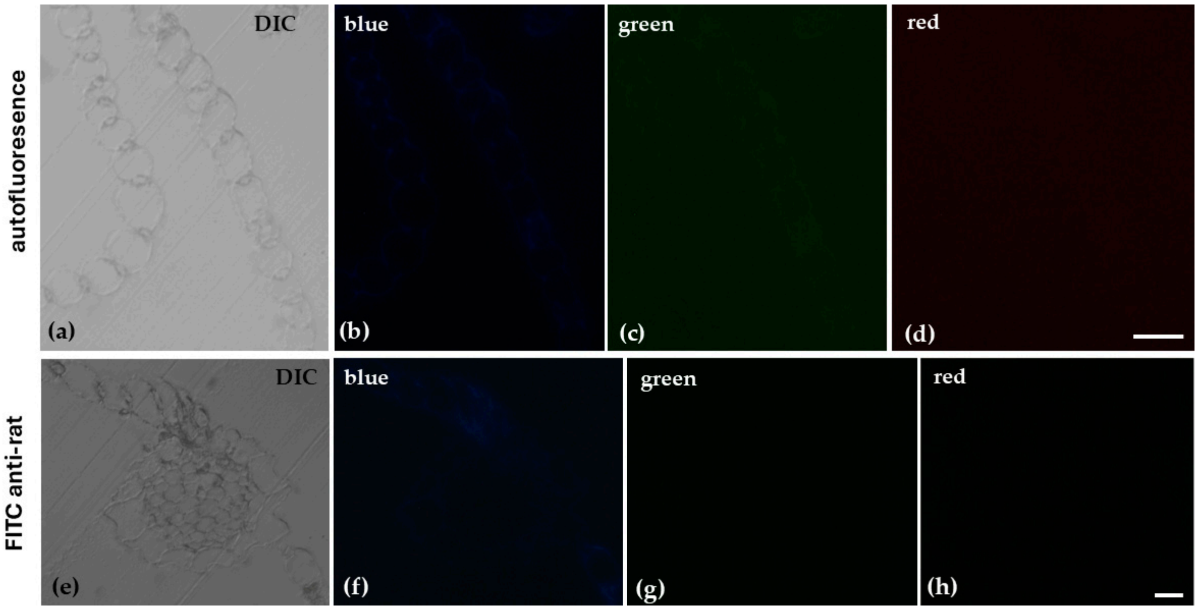

Figure S1. (a-d) *Sphagnum compactum* leaf cross section observed under Differential Interference Contrast (DIC) optics (a) and autofluorescence imaging (b-d) using blue, green, and red epifluorescence filters. No fluorescence is detected. (e-h) *Sphagnum compactum* stem and leaf cross section subjected to the immunolabeling protocol, applying only the secondary antibody (FITC anti-rat) as a control. Images show DIC optics (e) and fluorescence microscopy (f-h) under blue, green, and red epifluorescence filters, respectively. No fluorescent signal is observed. Scale bars: 20 μm.

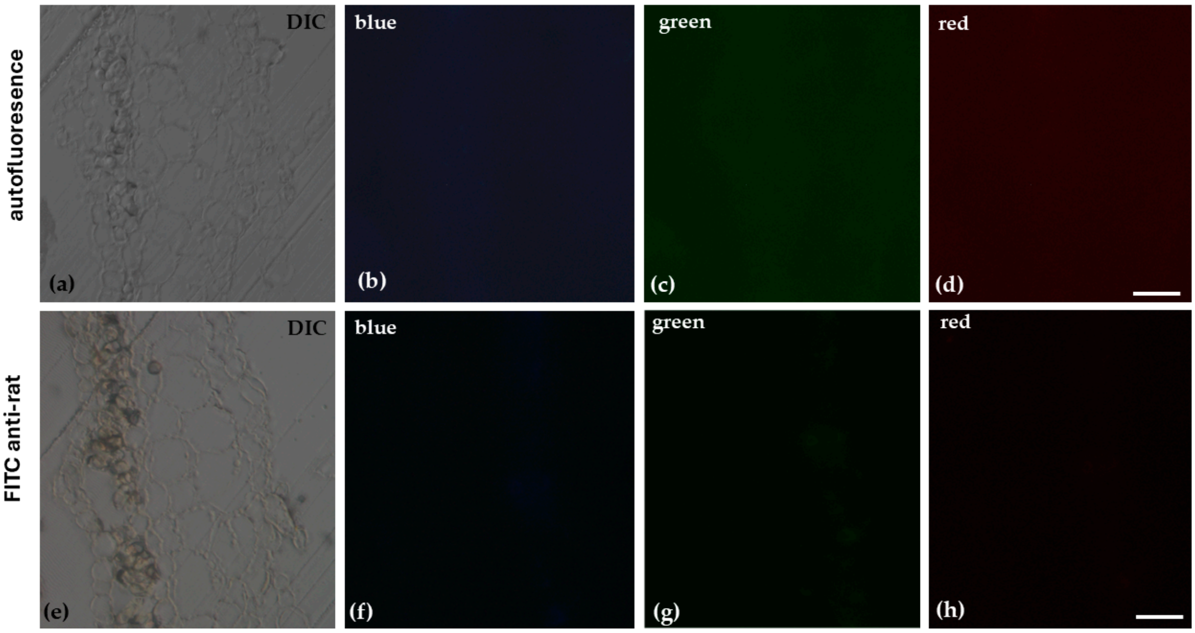

Figure S2. (a-d) *Marchantia polymorpha* thallus cross section observed under Differential Interference Contrast (DIC) optics (a) and autofluorescence imaging (b-d) using blue, green, and red epifluorescence filters. No fluorescence is detected. (e-h) *Marchantia polymorpha* thallus cross section subjected to the immunolabeling protocol, applying only the secondary antibody (FITC anti-rat) as a control. Images show DIC optics (e) and fluorescence microscopy (f-h) under blue, green, and red epifluorescence filters, respectively. No fluorescent signal is observed. Scale bars: 20 μm.
